# Common Protective Strategies in Neurodegenerative Disease: Focusing on Risk Factors to Target the Cellular Redox System

**DOI:** 10.1155/2020/8363245

**Published:** 2020-08-01

**Authors:** Patrizia Hrelia, Giulia Sita, Marina Ziche, Emma Ristori, Angela Marino, Marika Cordaro, Raffaella Molteni, Vittoria Spero, Marco Malaguti, Fabiana Morroni, Silvana Hrelia

**Affiliations:** ^1^Department of Pharmacy and Biotechnology, Alma Mater Studiorum, University of Bologna, 40126 Bologna, Italy; ^2^Department of Medical Science, Surgery and Neuroscience, University of Siena, 53100 Siena, Italy; ^3^Toscana Life Sciences, 53100 Siena, Italy; ^4^Department of Chemical, Biological, Pharmaceutical and Environmental Sciences, University of Messina, 98166 Messina, Italy; ^5^Department of Biomedical and Dental Sciences and Morphofunctional Imaging, AOU Policlinico Universitario “G. Martino”, 98125 Messina, Italy; ^6^Department of Medical Biotechnology and Translational Medicine, University of Milan, 20129 Milan, Italy; ^7^Department for Life Quality Studies, Alma Mater Studiorum, University of Bologna, 47900 Rimini, Italy

## Abstract

Neurodegenerative disease is an umbrella term for different conditions which primarily affect the neurons in the human brain. In the last century, significant research has been focused on mechanisms and risk factors relevant to the multifaceted etiopathogenesis of neurodegenerative diseases. Currently, neurodegenerative diseases are incurable, and the treatments available only control the symptoms or delay the progression of the disease. This review is aimed at characterizing the complex network of molecular mechanisms underpinning acute and chronic neurodegeneration, focusing on the disturbance in redox homeostasis, as a common mechanism behind five pivotal risk factors: aging, oxidative stress, inflammation, glycation, and vascular injury. Considering the complex multifactorial nature of neurodegenerative diseases, a preventive strategy able to simultaneously target multiple risk factors and disease mechanisms at an early stage is most likely to be effective to slow/halt the progression of neurodegenerative diseases.

## 1. Introduction

Neurodegenerative diseases define diversified chronic disorders related to the progressive motor, sensory, and perceptual dysfunctions which lead to cognitive and behavioural deficits. In these pathologies, the selective neuronal cell loss appears in the adulthood, within different areas of the brain [1]. Neurodegenerative diseases are usually divided into two main groups, chronic and acute disorders [2]. In particular, Alzheimer's disease (AD), Parkinson's disease (PD), Huntington's disease (HD), amyotrophic lateral sclerosis (ALS), and so forth, share a plethora of features like oxidative stress, glycation, abnormal protein deposition, inflammation, and progressive neuronal loss [3–5]. It is interesting to highlight that, several years later traumatic brain injury (TBI) or stroke, patients have shown an increased incidence of neurodegenerative chronic diseases [6–9]. In particular, after TBI, many patients show motor and cognitive manifestations similar to those observed in AD and PD patients [10–12]. During the last century, a growing research interest has been addressed to the identification of mechanisms and risk factors leading to the complex etiopathogenesis of neurodegenerative diseases, including not only genetic, vascular, and metabolic but also lifestyle-related factors, which often coexist and interact with each other [13–15].

In view of the complex multifactorial nature of neurodegenerative diseases, interventions that simultaneously target multiple risk factors and disease mechanisms at an early stage of the diseases are most likely to be effective. Among the matrix of factors which could delineate the possible pathogenesis of neurodegenerative diseases, aging is the primary risk, and also, cerebrovascular diseases, diabetes, and inflammation define steps in this inexorable complex cascade [16]. The effects of the different risk factors depend on the patient's age at treatment, indicating that the timing of preventive interventions needs to be carefully considered.

Inflammation is one of the key connectors linking vascular abnormalities and neurodegeneration. Indeed, inflammation, especially of the endothelium, is central to the initiation and progression of a broad spectrum of age-related neurodegenerative diseases [17], and it has been demonstrated to clearly affect the expression of Brain-Derived Neurotrophic Factor (BDNF) within the brain [18]. Neuroinflammation is a key factor in both acute and chronic conditions [19–21]. In the central nervous system (CNS), cellular infiltration in response to inflammation, infection, and injury is weaker and delayed than in other tissues, but microglia, and the expression and release of classical inflammatory mediators, such as acute-phase proteins, eicosanoids, complement, and cytokines, can be induced rapidly [22–24].

Moreover, redox signalling dysregulation has been recognized as a contributing factor in several age-related diseases and is responsible for endothelial dysfunction in the majority of pathophysiological conditions [25, 26]. Several studies established that radical detoxification pathways are key homeostatic mechanisms associated with vasoprotection in aging and chronic degenerative diseases [27–29]. In addition, oxidative stress is also correlated with the impairment of blood glucose regulation [30].

Nuclear factor (erythroid-derived 2)-like 2 (Nrf2) and nuclear factor-*κ*B (NF-*κ*B) are two interconnected master regulators of cellular responses to oxidative stress and inflammation, respectively [31]. Recently, several studies demonstrated that dysfunctions in redox homeostasis are a common mechanism in cardiovascular, neurological, and metabolic diseases [32, 33]. However, oxidative stress was hitherto not pharmacologically targetable, and the only strategy tested so far, using antioxidants, was unsuccessful or even harmful. Interestingly, small molecules, now become available, are able to interact with specific targets and useful for therapeutic proof-of-concept studies. In this view, the importance of investigating the complex interrelated molecular mechanisms behind neurodegenerative disease onset and progression appear undeniable. In an attempt to characterize the complex network of molecular mechanisms underpinning acute and chronic neurodegeneration, this review is focused on the disturbance in redox homeostasis, as common mechanism behind five pivotal risk factors: aging, oxidative stress, inflammation, vascular injury, and glycation.

## 2. The Role of Aging and Oxidative Stress in Chronic Neurodegenerative Diseases

AD is the leading cause of dementia worldwide, accounting for 60–70% of cases (http://www.who.int/mediacentre/factsheets/fs362/en/), although increasing evidence shows that mixed brain pathologies (AD and vascular) account for most dementia cases in the old age [34, 35]. Previous intervention efforts focused on the management of single risk factors with relatively modest findings.

Undoubtedly, aging is the primary risk factor for neurodegenerative diseases, and age-related changes in cellular function predispose to the pathogenesis of different pathological conditions, as AD. The EU population aged 65 and over is expected to double by 2030 and to triple by 2050 [36]. Aging not only makes patients more susceptible to neurodegenerative diseases but also impairs self-repair abilities. The number of people living with neurodegenerative diseases worldwide is currently estimated at 50 million (http://www.who.int/mediacentre/factsheets/fs362/en/).The economic-social burden of neurodegenerative diseases is devastating not only for the patients but also for their families and caregivers. Indeed, the huge cost of the diseases will challenge health systems to deal with the predicted future increase of prevalence. Thanks to the advances in molecular biology, our knowledge of aging and cognitive decline constantly increases. Many signalling pathways involved in the regulation of aging and lifespan have been identified, and recent studies have demonstrated the involvement of these signalling pathways in age-related cognitive decline [37, 38]. These pathways may represent important targets to develop novel and effective disease-modifying drugs to treat, delay, or prevent age-related neurodegenerative diseases.

Unfortunately, to date, no effective treatments are available to slow or stop the death and malfunction of neurons in the brain that cause disease symptoms and make the disease fatal. In this view, discovering new strategies and drugs to slow down the onset and the progression of neurodegenerative diseases is a primary goal, and it could have significant social and economic impacts. *β*-Amyloid (A*β*) plaque depositions and neurofibrillary tangle (NFT) accumulation not only are referred to as neuropathological hallmarks of AD but also have been widely implicated and described in the healthy aging process [39–41]. The chronic increase of oxidative stress has been recognized as a key contributing factor in aging and in several age-related diseases. Indeed, the “oxidative stress theory of aging” considers the functional impairments associated with aging, due to the accumulation of oxidative damage to lipids, DNA, and proteins by reactive oxygen species (ROS) and reactive nitrogen species (RNS). However, the exact mechanism by which oxidative stress induces aging is still not defined. Perhaps, the enhanced levels of ROS and RNS lead to cellular senescence, which involves the secretion of soluble proinflammatory factors and degradative enzymes [42]. In this area, S-nitrosylation, a covalent reaction of a nitric oxide (NO) group with a reactive cysteine thiol group on target proteins, has emerged as the principal mechanism exerting NO bioactivity [43]. S-Nitrosylation regulates protein function and can mediate either protective or neurotoxic effects depending on the action of the target protein [44]. Under physiological conditions, NO production induced by GMPc activation generates mitochondrial biogenesis through peroxisome proliferator-activated receptor *γ* (PPAR*γ*) coactivator. In contrast, increased nitrosative stress can result in defects in mitochondrial function. For example, S-nitrosylation affects mitochondrial respiration by inhibiting complexes I and IV [45]. Interestingly, Cho et al. demonstrated that S-nitrosylation of Drp1 mediates A*β*-induced disruption of mitochondrial dynamics, contributing to synaptic injury and neuronal damage [46]. Thus, protein modifications produced by RNS may impair mitochondrial health and further induce synaptic dysfunction and neuronal death. Indeed, another feature of AD brains is mitochondrial dysfunction [47, 48], characterized by an increase in mitochondrial membrane permeability and loss of membrane potential and associated with the release of cytochrome c [49, 50]. Interestingly, Antequera et al. [51] found a reduction in the expression levels of mitochondrial complexes I and III. They speculate that this mitochondrial dysfunction is probably because A*β*-related mitochondrial dysfunction is exacerbated by aging and may be one of the mechanisms explaining the pronounced accumulation of AD pathology with aging. The hypothesis is that the increasing levels of A*β* and the aging process in AD patients could be considered responsible for the senescent phenotype involving also endothelial cell (EC) dysfunction and characterized by increased oxidative stress [42]. In a recent study, Zhu et al. showed that in an aging mouse model (SAMP8), the cognitive impairment, inflammation, and oxidative stress were efficiently counteracted by the treatment with ligustilide, the most biological active component present in *Angelica sinensis*, a perennial plant that belongs to the Umbelliferae family [52]. Several studies have shown the ability of ligustilide to cross the blood-brain barrier (BBB) and to reach the CNS where the active could exert its antiapoptotic and antioxidative effects [53, 54]. The fundamental role of oxidative stress in neurodegenerative disorders is recognized, and, also in the early stages, it is possible to observe a significant increase of ROS production [55]. When this phenomenon is efficiently reduced, also the cognitive impairment and the inflammatory processes are successfully counteracted [56, 57]. Indeed, there is a close relationship among oxidative stress, aging, and inflammation.

During aging, the chronic oxidative stress enhances the loss of homeostasis, involving in particular the regulatory systems, as the immune response. This condition activates the inflammation that, in turn, increases oxidative stress generating a vicious circle [58]. A recent study has shown that increased levels of biomarkers for oxidative stress are related to high levels of inflammatory cytokines, and both are ascribed to poor cognitive performance in aged patients [59]. Several studies have shown that cognitive decline is slower when endogenous antioxidant systems, as glutathione peroxidase (GSH-Px), are high. On the contrary, high levels of GSH accelerate cognitive impairment in aged patients [55, 60]. This is a controversial event, because GSH is known as an endogenous protection against intracellular oxidative stress. An explanation could be that, as GSH is a substrate of GSH-Px, the increasing of GSH levels may be due to the increase of oxidative stress related to the reduction of GSH-Px activity [61]. The increased level of oxidative stress was observed also in human peripheral blood mononuclear cells isolated from individuals with mild cognitive impairment and from 3-month-old 3xTg-AD male mice, which was probably due to the increased levels of the Nrf2 and reduced superoxide dismutase 1 (SOD1) mRNA in the brain cortex [62]. It is known that Nrf2 is referred to as the principal regulator of the cellular response to oxidative and toxic insults, modulating the expression of hundreds of genes responsible for the immune and inflammatory responses, cellular metabolism and metabolic regulation, and even cognitive dysfunction and addictive behaviour [63]. The regulation of Nrf2 is complex and controlled not only by the repressor protein Kelch ECH associating protein 1 (Keap1) but also by other signalling pathways, including glycogen synthase kinase 3 (GSK-3), NF-*κ*B, NOTCH, and AMP kinase [60, 64, 65]. Due to the role of Nrf2 deregulation in neurodegenerative diseases, Nrf2 inducers are currently under investigation. The AT-Nrf2-knockout mouse model, which combines amyloidopathy and tauopathy with Nrf2 deficiency, presents increased markers of oxidative stress and neuroinflammation in the brain tissue compared to wild-type mice [66]. Furthermore, young adult AT-Nrf2-knockout mice have shown deficits in spatial learning and memory and reduced long-term potentiation. Transcriptomic analysis has shown that Nrf2-knockout mouse brains share 7 and 10 of the most dysregulated pathways with aging humans and AD brains, respectively [66].

## 3. Neuroinflammation and Aging: Role of Acute Injury and Impact on Neurotrophins

Among the principal causes of acute brain injury, TBI and stroke are the most relevant. TBI is a highly complex disorder caused by both primary and secondary injury mechanisms [67, 68]. Primary injury mechanisms result from the mechanical damage of neurons, axons, glia, and blood vessels as a result of shearing, tearing, or stretching. Secondary injury mechanisms include a wide variety of processes such as depolarizations and disturbances of ionic homeostasis [69], release of neurotransmitters (e.g., glutamate excitotoxicity) [70], mitochondrial dysfunction [71], neuronal apoptosis [72], lipid degradation [73], and initiation of inflammatory and immune responses [7, 74].

Likewise, strokes can be classified into two main types: ischemic or haemorrhagic. In the first case, the neurological dysfunction is caused by focal cerebral, spinal, or retinal infarction. The haemorrhagic stroke can be classified as subarachnoid haemorrhage (SAH), with haemorrhage from a cerebral blood vessel, aneurysm, or vascular malformation located into the subarachnoid space, or as intracerebral haemorrhage (ICH), when a weakened blood vessel within the brain bursts, allowing blood to leak and increasing intracranial pressure, causing damage to the brain cells surrounding the blood [75–77].

Posttraumatic neuroinflammation is characterized by oxidative stress, glial cell activation, leukocyte recruitment, and release of inflammatory mediators [74], as hereafter reported. High ROS levels cause lipoperoxidation of cell membrane, leading to dysfunction of mitochondria and oxidizing proteins [78]. After injury, endogenous inflammatory responses are activated to protect the damaged area from invasion of pathogens and to restore injured cells. In this condition, the complement system is activated, followed by the invasion of monocytes, neutrophils, and lymphocytes through the BBB [79], with consequent production of prostaglandins, proinflammatory cytokines, free radicals, and several inflammatory elements. Microglia are the primary innate immune cells in the CNS and represent the first line of defence following brain injury [80]. On the other hand, when microglia become overactivated or reactive, they can induce detrimental neurotoxic effects by releasing multiple cytotoxic substances, including proinflammatory cytokines and oxidative metabolites [81]. Further, the release of proinflammatory cytokines and other soluble factors by activated microglia can significantly influence the subsequent activation of astrocytes [82].

Upon activation, astrocytes upregulate several neurotrophic factors (e.g., BDNF) that protect against cell injuries [83]. In addition, astrocytes play a crucial role in regulating excitotoxicity by reducing neuronal glutamate levels [84]. These alterations may lead to secondary neurological disease, such as ischemia and epilepsy [85]. After injury, neutrophils are the first immune cells that undergo conformational changes and migrate through the endothelial vessel wall to invade the damaged tissue [86]. Following an ischemic injury, neutrophils cause secondary injury by releasing proinflammatory factors, ROS, proteases, and matrix metalloproteinases (MMPs) [87]. These toxic factors impair EC membrane and basal lamina leading to the increase of BBB permeability [23]. In addition, leukocytes potentiate ischemic injury blocking erythrocytes' flow and then activating the production of proteases, MMPs, and ROS that can significantly damage blood vessels and brain tissues. Finally, infiltrated leukocytes infiltrated further exacerbate neuronal injury by activating proinflammatory factors in and around the penumbra and the infarct core [23, 88, 89]. Cytokines upregulate the expression of cell adhesion molecules (CAM) [90, 91], as the intracellular adhesion molecule 1 (ICAM 1) in the ischemic core which leads to BBB disruption [23]. The three major proinflammatory cytokines are tumor necrosis factor-alpha (TNF-*α*), interleukin 1*β* (IL-1*β*), and IL-6 that contribute to the inflammatory response after brain injury [92, 93]. Under certain stimuli, TNF-*α* is synthesized and released by astrocytes, microglia, or neurons and is involved in the BBB permeability and in the modulation of synaptic transmission and plasticity [94–96]. After the formation of an inflammasome, IL-1*β* can activate NF-*κ*B via toll-like receptors (TLRs) allowing the nuclear factor to transactivate genes associated with cytokines, chemokines, and other proinflammatory mediators. In addition, IL-1*β* can prime the endothelium for increased leukocyte adherence and edema formation [97]. Additionally, Yang and colleagues demonstrated that IL-6 serves as an amplification signal for the inflammatory response and motor coordination deficits after brain injury [98].

Age at injury is likely to influence the way the brain is able to repair itself as a result of developmental status, extent of cellular senescence, and injury-induced inflammation [99–102]. Hoane and colleagues and Sohrabji showed that aging increased tissue loss compared to young animals following TBI and also state that aging is the principal risk factor for ischemic stroke [103, 104]. This is probably due to the functional changes that happened in the BBB as a result of brain injury, including decreased trafficking of peripheral immune cells into the brain parenchyma and increased oxidative stress and inflammatory mediator release that lead to an amplification of the inflammatory response in the injured brain [105]. For this reason, the understanding of cell-specific changes in an aging brain will be critical for the development of next-generation drug therapies.

As the molecular mechanism of aging in mice is similar to that in humans [95], mouse models have been often used in the field of neurodegenerative diseases associated with aging [106]. In particular, studies have been conducted to better focus on major risk factors for PD, reportedly associated with aging [107]. In this regard, Crupi et al. already reported about PD modelled on old mice by 1-methyl-4-phenyl-1,2,3,6-tetrahydropyridine (MPTP). In particular, old MPTP-intoxicated mice (21 months old) and young MPTP-intoxicated mice (3 months old) were both subjected to behavioural testing and brain processing eight days after MPTP administration [108]. The authors demonstrated a more significant nigrostriatal dopamine (DA) degeneration than that observed in young MPTP-treated mice. Moreover, anxiety-like behaviour was more evident in MPTP-treated old mice. In this context, the aim of the authors was to define a time window for applying therapeutic treatment to effectively counteract neurodegenerative processes associated with age-related diseases. As a matter of fact, current therapies do not address neuroinflammation but, though neuroinflammation may worsen PD disease progress, they are focused on ameliorating the symptoms of DA loss rather than the mechanisms underlying DA neuron damage [109].

As neurodegenerative diseases, associated with inflammation and oxidative stress, may develop as a consequence of brain trauma, studying the onset of neurodegeneration in MPTP mouse models, in young and aged animals, can be considered a good basis. In this context, Calabrese et al. state that peripheral and/or central inflammatory stimuli, affecting the brain, could induce inflammatory changes leading to PD symptoms and progression [107].

The abnormal neuroinflammatory response and oxidative stress may have a detrimental impact on neuroplasticity, the ability of the brain to perceive and respond to an external or internal stimulus through an adaptive mechanism, which is compromised in several neurodegenerative disorders [110]. This CNS capability to shape its structure and function for a proper coping relies on the integrated involvement of different molecular systems, among which the neurotrophic factors plays a crucial role. Indeed, it is well known that the diversity and specialization of the CNS resident cellular populations are due to many complex processes. Proliferation, differentiation, growth, migration, synaptic formation, and modification are mainly carried on by neurotrophic factors, in particular by neurotrophins (NTs). NTs are a group of polypeptide growth factors secreted by different brain cell populations, such as microglia cells, oligodendrocytes, astrocytes, and neurons. The NT family comprehends different but similar polypeptides: the nerve growth factor (NGF), BDNF, NT-3, and NT-4/5, as well as the more recent NT-6 and NT-7. Their activity is mediated by the binding to specific transmembrane receptors, the tropomyosin receptor tyrosine kinases (Trk receptors) and the p75 NT receptor. NTs have different binding affinities for specific receptors: NGF binds to TrkA, BDNF and NT-4 to TrkB, and NT-3 to TrkC, whereas all four NTs can bind to the p75 receptor. Furthermore, the association of p75 with Trk receptors can increase the selective affinity of the second ones for each respective NT [111, 112]. Nowadays, the role of NTs for the survival of developing neurons is well consolidated [113, 114]; however, in the last decades, the focus of the research has moved on their function as mediators of neural and synaptic plasticity in the adult brain. In particular, BDNF has emerged for its role in a wide range of neurophysiological processes, peculiar activity-dependent regulation, and because of its abundance in brain regions involvement in neuroplasticity throughout the lifespan. The wide spectrum of activity in which BDNF is involved relies to its complex genetic structure that has been characterized in detail [115, 116]. BDNF gene contains multiple promoters that drive the expression of several transcripts bearing different noncoding exons. Interestingly, different isoforms of BDNF are expressed in different subcellular compartments; for example, exon IV mRNAs have been detected in the soma and dendrites while exon III expression is restricted to the cell body [117]. It is important to note that the transcripts that target the dendritic area may promote fast local translation of the pro- and mature BDNF, producing an effect strictly linked to the synaptic structure and activity [118, 119]. The synthesis of the mature BDNF is likewise a complex process, involving different precursor isoforms and different possible pathways to reach the mature form. The pro-BDNF protein, indeed, can be cleaved both in the intracellular space, in the intracellular secretory vesicles, or after secretion, through distinct mechanisms. Pro-BDNF is also an active precursor, which is able to bind the p75 neurotrophin receptor and the sortilin receptor, while mature BDNF binds p75 receptor and, preferentially, TrkB [120]. Upon binding with BDNF, TrkB initiates dimerization and autophosphorylation. Once phosphorylated, TrkB activates a series of intracellular pathways: the phosphatidylinositol 3-kinase/protein kinase B- (PI3K/Akt-) related pathways, which exert antiapoptotic and prosurvival activities and modulate N-methyl-D-aspartate receptor- (NMDAR-) dependent synaptic plasticity [121–123]; the PI3K/Akt/mammalian target of rapamycin (mTOR) cascade that, through regulation of protein synthesis and cytoskeleton development, enhances dendritic growth and branching [124, 125]; the mitogen-activated protein kinase (MAPK)/Ras signalling cascade that regulates protein synthesis during neuronal differentiation [126]; and many others.

Given the crucial physiological role that BDNF exerts through the above-described mechanisms on several processes known to be compromised in neurodegenerative disorders, such as neuronal survival and cognition, several clinical and preclinical studies have investigated the impact of the risk factors for these diseases on BDNF function, in particular focusing on the influence of aging. The obtained results clearly underline a relationship not only between aging and deficit in neuroplasticity but also between BDNF alteration and frailty, the fragility that may underline neurodegenerative diseases in the elderly [127]. Indeed, it is important to note that some individuals are able to reach advanced age with the cognitive functions mainly intact whereas others develop a condition of frailty, characterized by an increased general vulnerability probably due to microtraumas and detrimental events accumulated during life. Furthermore, even the high-functioning elder people who experience an acute injury (such as TBI or stroke), a stress, or an infection become at higher risk to develop a transient or permanent cognitive impairment, which may in turn result in dementia and other symptoms of neurodegenerative diseases. To the current knowledge, the cognitive impairment observed in the aged population is due—at least in part—to structural and physiological changes in the brain. During aging, these processes undergo a physiological decline, and structural changes in neurons and spines as well as alterations in neurotransmitter receptor expression and changes in electrophysiological properties occur, causing an increased vulnerability to neurobiological diseases [128].

In the attempt to explain what is observed during aging, a negative correlation between BDNF serum levels and aging has been found in healthy subjects [129]. Moreover, the hippocampal volume of 142 healthy subjects between 59 and 81 years old has been measured and correlated with serum BDNF levels and memory performances finding that increasing age was associated with smaller hippocampal volumes, reduced levels of serum BDNF, and poorer memory performances [130]. Furthermore, a postmortem study on healthy subjects aged between 16 and 96 years confirmed the negative correlation between BDNF and age in the orbitofrontal cortex and showed that the expression of synapse-related genes belonging to the BDNF network was downregulated with age as well [131]. Among the mechanisms that may affect the BDNF system during aging, an abnormal activation of the immune/inflammatory system is thought as an important candidate. Indeed, it is well known that the inflammatory response may affect neuroplasticity during development and adulthood [132]. Moreover, during aging, the immune system undergoes a dysregulation that leads to a chronic systemic inflammation, with increased levels of cytokines, chemokines, proinflammatory enzymes, and transcription factors [133, 134].

**Figure 1:**
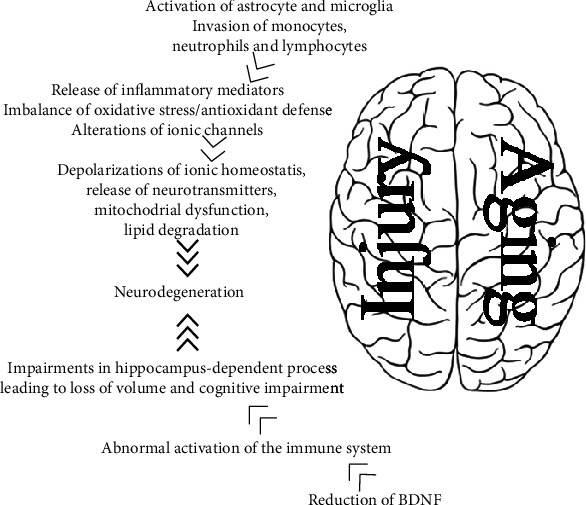
Contribution of inflammation, oxidative damage, and reduction in NT levels to neurodegeneration in aged brain after injury.

The “inflammaging” state does not rule out the brain, as the peripheral circulating small molecules—such as cytokines—can penetrate the CNS through the BBB inducing a cerebral state of neuroinflammation that can be further amplified by the activation of microglia [135]. In this context, it has been demonstrated that the activity of macrophages is specifically modified during aging, suggesting also a possible role for oxidative stress [136, 137]. Under physiological conditions, microglia cells are in an apparent resting state in which they actively survey the CNS environment, ready to intervene when a detrimental stimulus occurs. Specifically, they undergo the activation state, with morphological changes and production of cytokines and of proliferative and macrophagic factors [138] following—when the threat (infection or damage) has been removed—to another state, characterized by a gene profile able to promote tissue repair and reconstruction, through the production of anti-inflammatory cytokines, growth factors, and NTs such as BDNF [139]. During aging, microglia cells undergo a series of modification such as telomere shortening and cellular dystrophy, which lead to its senescence. In a postmortem study, Streit et al. observed significantly more dystrophic changes in microglia of aged individuals (68-year-old) than in the younger ones (38-year-old) [140]. Interestingly, it has been described that dystrophic or senescent microglia might undergo age-dependent degeneration losing its neuroprotective functions, thus increasing the risk of developing a neurodegenerative disease [141]. Using flow cytometry in mice, Ritzel et al. identified a significant population of side scatter-high microglia in the aged brain that display functional abnormalities when compared to young microglia, including higher production of ROS and proinflammatory cytokines, increased mitochondrial content, and poor phagocytic ability [142]. Furthermore, aged microglia cells adopt a proinflammatory state due to a decrease in the resting signalling by neurons and astrocytes [143]. As a result, external stimuli (e.g., stress, trauma, and infection) can easily switch the aged brain into a state of mild chronic neuroinflammation, making the brain more prone to apoptotic signalling [144–146], leading to loss of volume and cognitive impairment [147] (Figure 1).

In particular, preclinical studies demonstrated that elevated hippocampal levels of IL-1*β* impair the performances in behavioural paradigms commonly used to examine hippocampus-dependent memory [148, 149]. Numerous studies in rodents confirmed these observations, demonstrating impairments in hippocampus-dependent contextual tasks following intraperitoneal (i.p.) [150] or intrahippocampal injection of IL-1*β* [151] and elevations in endogenous IL-1*β* evoked by infections [150–152] or psychological and physical stressors [153, 154].

As previously mentioned, this aging-dependent low-grade chronic inflammation is thought to contribute to the reduction of BDNF levels observed in the older population. Guan and Fang, in a preclinical study, demonstrated that a peripheral injection of lipopolysaccharide (LPS), a strong cytokine inducer, causes a reduction of the protein levels of BDNF in different cortical regions as well as in the hippocampus of adult rats [155]. These observations have been confirmed also in mice, where reduced protein levels of pro-BDNF, mature BDNF, and BDNF mRNA levels have been found in synaptosomes three days after the LPS i.p. injection [156]. A similar result was observed in aged animals five days after the inflammatory challenge. Specifically, Cortese et al. exposed aged rats to *E. coli* i.p. administration to induce a peripheral inflammatory response finding reduced levels of mature BDNF and TrkB activation in comparison to aged rats treated with vehicle as well as to young rats exposed to *E. coli* [157]. Furthermore, the central administration of a receptor antagonist for IL-1 simultaneously to the *E. coli* injection was able to block the observed reduction of BDNF [158], as well as the associated long-term memory impairment caused by the *E. coli* injection [159]. In line with these observations, the infusion of the proinflammatory cytokine IL-1*β* into the hippocampus decreased the induction of BDNF gene expression induced by contextual fear conditioning [158].

## 4. Mechanisms of Neurodegeneration Associated with Endothelial Cell Dysfunction

Vascular risk factors such as age, diabetes, hypertension, and hypercholesterolemia often overlap with neurodegenerative risk factors in older patients, and vascular dysfunction is recognized as a determinant in several neurodegenerative diseases such as AD, cerebral amyloid angiopathy (CAA), PD, and ALS [160–163]. The BBB integrity, as part of the neurovascular unit (NVU), is essential to maintain adequate brain perfusion and brain functionality and to preserve normal neurological functions. Oxidative stress plays a critical role also on pathological BBB impairment and on the cerebrovascular dysfunction observed in neurodegenerative diseases.

AD is characterized by an excessive deposition of A*β* protein that destabilizes vascular integrity, promoting vascular leakage. Loss of vessel integrity manifests with EC detachment from basal membrane, double-barreling of the vessel walls, and aneurysm formation. These events often result in blood extravasation to the perivascular space and in the initiation of an inflammatory response, which characterizes neurodegenerative diseases. Several studies have clearly shown that pathological concentrations, in the range of micromolar, of different A*β* peptides, in particular the shorter vasculotropic A*β*_1-40_ variant and the A*β* mutants, are associated with distinct hereditary phenotypes of CAA and impair angiogenesis and vascular maintenance by increasing cellular oxidative stress. The vascular damage induced by A*β* includes alteration of vascular tone, impairment of vascular remodelling, and loss of barrier functions, as well as suppression of the intrinsic angiogenic properties of the endothelium.

Donnini et al. demonstrated that the A*β*_1-40_ peptide and its Dutch E22Q variant cause a premature senescent phenotype in ECs in both zebrafish embryos and human ECs [164]. A*β*_1-40_ peptide also causes mitochondrial impairment and reduces the aldehyde dehydrogenase-2 (ALDH2) detoxifying enzymatic activity in ECs, resulting in cell membrane disorganization and permeability defects [165]. Similarly, the A*β*_1-42_ peptide has been reported to induce endoplasmic reticulum stress in rat brain ECs, subsequently leading to vascular derangements [166]. The molecular mechanisms of these multiple A*β*-induced effects on ECs are complex and may include direct and indirect interaction with angiogenic growth factors, including vascular endothelial growth factor (VEGF) and fibroblast growth factor-2 (FGF-2).

FGF signalling is a prominent pathway involved in the maintenance of integrity in quiescent vasculature. Solito et al. showed that A*β*_1-40_ and its arctic E22G and Dutch E22Q variants downregulate FGF-2 production and FGF-2-induced Akt activation. Moreover, A*β*_1-40_ and its variants inhibit FGF-2 binding to heparin and FGF receptor 1 phosphorylation, both *in vivo* and *in vitro* [167, 168]. Of note, the disruption of vascular integrity by the A*β*_1-40_-induced deregulation of the FGF-2 signalling pathway can be rescued forcing overexpression of FGF-2 in ECs. Indeed, ECs overexpressing FGF-2 displayed extraordinary resistance to A*β*_1-40_-induced injuries. The FGF-2 mechanism responsible for reversing damages involves the downstream enhancement of Akt and the endothelial nitric oxide synthase (eNOS) activation [167].

Several studies showed that A*β* also affects VEGF signalling. The VEGF receptor-2 mRNA and the protein levels are significantly decreased after A*β*_1-40_, both in EC and in the brains of AD mouse models [169]. Patel et al. showed that A*β*_1-42_ inhibits VEGF-induced migration of ECs, competing with the VEGF for the binding with its receptor VEGFR [170]. Moreover, cell culture studies revealed that A*β* at pathological concentrations acts as a VEGF antagonist, inhibiting VEGF-induced tyrosine phosphorylation of VEGFR-2, as well as VEGF-stimulated phosphorylation of Akt and eNOS in ECs [170–172].

The A*β* precursor protein (APP) is expressed in several tissues and cells, such as the brain, kidney, platelets, and vascular endothelium of cerebral and peripheral blood vessels. Interestingly, several studies showed a vascular function of APP and/or A*β* on ECs [173]. In cultured cerebral and peripheral ECs, nanomolar (nM) concentrations, similar to the physiological level of either A*β*_1-40_ or A*β*_1-42_ peptides, promote angiogenesis by increasing growth, migration, and tube branching [174, 175]. Thus, oxidative stress is induced in ECs by high concentrations of A*β* peptide, which accumulates in the vessels of BBB and in the brain parenchyma. However, physiological levels of A*β* are also required for the endothelial homeostasis, and increasing evidence highlights in several organs the importance of APP and its metabolites in supporting the function of the vascular tissue [173, 176]. The evidence that clinical trials aimed at targeting A*β* with immunotherapy have failed and, in some cases, have been harmful recalls the physiological role of A*β* and its precursor protein APP in the vasculature. More studies are needed to elucidate why ECs express high levels of APP and A*β* and what the functional role of these molecules is at a vascular level.

As we know, oxidative stress and mitochondrial dysfunctions are key actors in neurodegenerative disease. The mitochondrial enzyme ALDH2 has been shown to have a critical role in the neurotoxic mechanisms of these pathologies [177–179]. The mitochondrial disorder may promote the production of ROS, which increases the susceptibility of the cell to oxidative stress. One of the consequences of excessive oxidative stress is the overproduction of toxic aldehydes by lipid peroxidation from the mitochondrial membranes. Reactive aldehyde accumulation may inhibit ALDH2 and trigger mitochondrial dysfunction leading to a higher aldehyde-induced damage in both vasculature and neural tissues. The ALDH superfamily plays a crucial role in many biological processes including development and detoxification pathways in the organism [180]. In particular, mitochondrial ALDH2 is crucial in the oxidative metabolism of toxic aldehydes in the brain, such as catecholaminergic metabolites (DOPAL and DOPEGAL) and 4-hydroxy-2-nonenal (4-HNE), the principal product of the lipid peroxidation process [178]. Recent studies have demonstrated that inhibition of ALDH2 activity significantly impairs EC functions, promoting senescence [181–183]. Lack of ALDH2 activity reduces cell proliferation and migration and increases cellular permeability in ECs. Although the mechanisms of action has not been fully elucidated, these studies suggest that the accumulation of endogenous reactive aldehydes such as 4-HNE and ROS production are the main causes of endothelial dysfunction [181, 182].

In AD and PD, the increase of oxidative stress, in part due to the formation of A*β* plaques and NFTs, can also be attributed to a failure of the detoxifying activity of ALDH2. This hypothesis is supported by the correlation between ALDH2 loss-of-function mutations and a higher incidence of AD [180]. Moreover, ALDH2 knockout mouse models exhibit both neuronal and vascular pathological changes associated with AD [183, 184]. In turn, A*β* peptide toxicity can also impair mitochondrial ALDH2 activity [165]. Interestingly, this study shows that activation of ALDH2 has a protective role in endothelium against A*β*_1-40_ insult [165]. Treatment with ALDH2-specific-activator, Alda-1, significantly protects mitochondria function and reduces neuronal cell death in animal models of parkinsonism [178, 180, 185]. Due to its crucial role in maintenance of mitochondrial normal function, the use of ALDH2 activators would protect both vessels and neurons from neurotoxicity; thus, ALDH2 activation may represent a therapeutic target to treat neurodegenerative diseases.

## 5. Advanced Glycation Endproducts Mediated Neurotoxicity and Their Influence on Redox Metabolism

Neurodegeneration-mediated neurotoxicity can be induced by glycation reactions. Early glycation adducts mainly consist in Amadori products generated by the rearrangement of a Schiff base, resulting from the reversible reaction between a carbonyl group and protein amino group, mainly from lysine or arginine residues [186]. Even though the formation of Schiff bases is a reversible process, early glycation adducts can further rearrange through cyclization, oxidation, dehydration, or condensation reactions, leading to irreversibly bound adducts known as advanced glycation end-products (AGEs) [187, 188] often responsible for protein cross-links [189]. Since glycation is a nonenzymatic process, proteins characterized by a slow turnover are those that more easily accumulate AGEs [187]. In human tissues, AGE formation was first studied in relation to high blood sugar levels and diabetes, but more recently, other compounds such as glyceraldehyde, glycolaldehyde, glyoxal, and methylglyoxal have been recognized responsible for glycation reactions [190].

Methylglyoxal (MG), an α-ketoaldehyde, can occur as glycolysis by-product, but it is also present in foods (especially cooked and baked), beverages (mainly those fermented), and cigarette smoke, and it is considered the most potent precursor of AGE formation [191, 192]. In fact, it results 20,000 times more reactive than glucose in glycation reaction [193]. More than 20 different AGEs have been identified in foods and in human tissues. The most important ones are represented by pyrraline, pentosidine, carboxymethyl-lysine (CML), carboxyethyl-lysine (CEL), and methylglyoxal-lysine dimer (MOLD) [194, 195]. Due to MG and other carbonyl reactivity and toxicity, eukaryotic organisms have developed specific enzymes to detoxify them. The glyoxalase system, in fact, is composed of glyoxalases I and II and combines *α*-ketoaldehydes to GSH to produce D-hydroxyacids [196]. Other enzymes and proteins contribute to counteract glycation; indeed, fructosamine-3-kinase catalyses fructosamine phosphorylation determining protein deglycation [197], and aldose reductase contributes to *α*-oxoaldehyde reduction [198].

Beside diabetic complications, AGE accumulation in blood and tissues has been related to many chronic and degenerative diseases, such as neurodegenerative and cardiovascular diseases, atherosclerosis, and cancer, to induce cell signalling impairment, oxidative stress, and inflammation, as well as protein aggregation and cross-links [16]. In this context, AGE accumulation, oxidative stress, and inflammation are related to AGE ability to bind specific receptors called RAGE. Indeed, the activation of the AGE pathway can deregulate gene transcription, the signalling between cells and the extracellular matrix, and blood proteins, leading them to bind to RAGE on macrophages that, in turn, increase the release of growth factors and proinflammatory cytokines [199].

RAGE belongs to the immunoglobulin superfamily and is found in numerous tissues such as cardiac, vascular, pulmonary, and brain tissues. Moreover, their expression increases during aging, cancer, cardiovascular diseases, AD, PD, and other neurodegenerative diseases [200–205]. Although they were first described as AGE binding receptors, many other ligands have been discovered, such as S100 family molecules as well as high-mobility group protein 1, known to be involved in inflammation and A*β* aggregation processes [206–208].

As soon as AGEs and other ligands accumulate, RAGE expression is induced [209] and elevated levels have been described in all the aforementioned pathological conditions and aging [203, 204].

AGE-RAGE binding activates numerous signalling pathways related to inflammation, oxidative stress, and apoptosis. RAGE activation has been demonstrated to induce NF-*κ*B, which in turn is responsible for an increased expression of proinflammatory cytokines [210] and for the activation of the MAPK signalling pathway through the phosphorylation of extracellular signal-regulated kinases (ERK1/2), p38, and JNK, leading to inflammation, proliferation, and apoptosis [211]. Moreover, AGE-RAGE binding results in oxidative stress by the induction of the prooxidant enzyme NADPH oxidase (NOX2) [212]. RAGE is not the only group of receptors able to bind AGEs. In fact, AGER1-3 are involved in AGE detoxification by binding them on the cell surface and regulating their endocytosis to reduce oxidative stress, RAGE, and inflammation [213]. Interestingly AGERs are downregulated in many chronic diseases and in the presence of high AGE concentration [214, 215].

**Figure 2:**
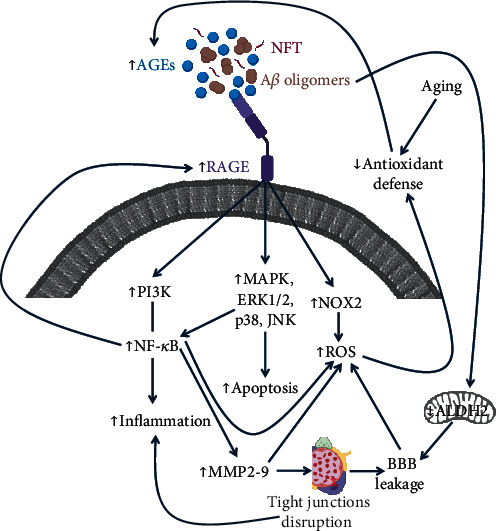
The vicious circle of the principal pathways involved in RAGE activation. AGE-RAGE binding activates different signalling pathways, in particular MAPK, PI3K, and NOX2, inducing inflammation, apoptosis, and oxidative stress. Moreover, the increased levels of NF-*κ*B induced the expression of RAGEs. The inflammatory response is also enhanced by the disruption of tight junctions at NVU that compromises also the BBB integrity. Oxidative stress is increased also by the high level of NF-*κ*B and by the consequent increment of MMP2-9. In aging, elevated ROS levels are not efficiently counteracted by endogenous antioxidative defences, and, as a consequence, AGE formation is increased. AGEs not only stabilize A*β* oligomers and NFT but also increase their formation. In addition, A*β* oligomers can also bind RAGE and activate the inflammatory/oxidative cascade. Finally, A*β* oligomers can trigger the impairment of mitochondrial ALDH2, leading to endothelial dysfunction and BBB leakage.

It is well known that AGE accumulation and oxidative stress play a central role in the pathogenesis of neurodegenerative diseases [216]. The brain, despite its high metabolic rate and oxygen consumption, is characterized by poor antioxidant defences; indeed, it presents weak expression of antioxidant enzymes as well as low levels of GSH and other antioxidants [217]. These aspects make the brain particularly prone to oxidative damage. In this context, AGEs play a dual role, since their formation is increased in oxidative conditions and because they promote oxidative stress [218]. AGE accumulation has been observed in brains affected by AD and PD as well as other neurodegenerative disorders [219]. Both A*β* plaques and NFT present AGE-induced protein cross-links, and A*β* aggregation is accelerated and stabilized in the presence of AGEs [220]. Besides their role in the stabilization of both A*β* and NFT, AGEs have been implicated also in their formation. Ko et al. demonstrated that AGEs induce APP expression, and also, glycated tau protein induces oxidative stress [221, 222]. Moreover, A*β* has been recognized as a RAGE ligand; A*β*-RAGE binding contributes to the disease progression by inducing neuroinflammation and oxidative stress [223]. AGEs have been demonstrated to contribute also to the aggregation of *α*-synuclein, a protein rich in lysine residues, to form Lewy bodies, a well-known biomarker of PD (Figure 2) [224, 225].

Beside pharmacological approaches, mainly focused on targeting RAGE [223, 226], a natural substance approach appears promising. Vitamin B1, being a coenzyme of transketolase, contributes to its activity and reduces the accumulation of glycolytic intermediates responsible for glycation's reactions [227]. Some flavonoids and other polyphenols have been proposed as safe candidates to delay the progression of AGE-mediated inflammatory diseases [228]. Some polyphenol-rich extracts, such as pomegranate, have been demonstrated to inhibit glycation or to trap MG in cell-free *in vitro* systems [229, 230].

Epigallocatechin gallate (EGCG) demonstrated to exert protective effects *in vitro* against AGE toxicity in neuronal cells. Lee and Lee showed, in SH-SY5Y cell culture, that 5-10 *μ*M of EGCG treatment counteracts oxidative stress, by inducing superoxide dismutase (SOD) and catalase (CAT), decreases MG levels and AGE formation, and downregulates RAGE expression [231]. In an *in vivo* rat model of AD, resveratrol (Res) has been found able to decrease RAGE expression at the hippocampus level and to exert anti-inflammatory effects as demonstrated by the decrease of NF-*κ*B protein expression [232]. Other studies have related Res anti-inflammatory properties to the induction of sirtuin 1 (SIRT1) protein, as demonstrated by Wang et al. in an *in vivo* rat model of AD. In SH-SY5Y cell culture, Res treatment counteracts oxidative stress and apoptosis induced by AGEs [201, 233, 234]. Recently, quercetin has been demonstrated to counteract dietary AGE-induced cognitive impairment in old ICR mice by inhibiting ERK1/2 and tau protein phosphorylation [235]. Angeloni et al. demonstrated that sulforaphane (an isothiocyanate from Brassica vegetables) protects SH-SY5Y neuronal cells against MG-induced damage by inhibiting the activation of caspase-3 enzyme and reducing the phosphorylation of ERK1/2, JNK, and p38 signalling pathways.

Moreover, sulforaphane was able to counteract oxidative stress and to increase intracellular GSH levels and the expression, and activity, of glyoxalase 1 [236, 237]. Bioactive substances from Olea europaea, such as oleocanthal and hydroxytyrosol, are able to counteract the glycation processes [238, 239]; moreover, oleocanthal treatment improves GSH intracellular content and counteracts oxidative stress in neuron-like cell culture [240]. Recently, Angeloni et al. analysed the relationship between oleocanthal and AD suggesting that, besides its effects to interfere with tau protein hyperphosphorylation and aggregation and its ability to induce A*β* efflux and clearance, it might counteract AD's progression by reducing glycation in the brain, thanks to its positive effect on the GSH level, and to its ability to decrease oxidative stress [241].

Regardless of neurodegenerative diseases, the possibility to counteract the glycation processes and AGEs' toxicity using bioactive substances has recently been corroborated by the fact that activators of the Nrf2 signalling pathway have been able to induce the expression of genes involved in carbonyl stress resistance. It has recently been shown in SH-SY5Y cell cultures that the activation of Nrf2 by carnosic acid causes an increase in the expression of factors involved in the synthesis of GSH and allows the detoxification of MG through the glyoxalase system, thus protecting the cells from MG-induced carbonyl stress [242].

In the light of these perspectives, it is now possible to speculate that the aforementioned protective effects of natural bioactive molecules against glycation and AGE's toxicity might be, at least in part, due to the modulation of Nrf2 as a key regulator of the inflammatory response and the oxidative damage related to neurodegeneration.

## 6. Conclusions

Neurodegenerative diseases have shown to share similar features. Although they have not been well characterized yet, oxidative stress, inflammation, excitotoxicity, and neuronal loss seem closely related in the evolution and progression of both chronic and acute conditions. Because of the high rate of oxygen consumption and the low detoxification mechanisms, the brain is an organ extensively exposed to oxidative stress [243]. The complex structure and functions of the brain still do not permit to clearly describe how neurodegeneration could evolve. The urgent need to study the intricate molecular mechanisms behind the onset and progression of neurodegenerative disease appears undeniable, in order to design more effective therapeutic strategies.

In this scenario, an intervention able to slow down or arrest the evolution of pathology could be the keystone in the treatment of these pathologies. A neuroprotective strategy interfering with the inflammatory response and oxidative stress may modulate positively the progressive impairment of the patients' quality of life. Neuroprotection could work in synergy with the endogenous defences, quenching ROS formation or restoring the antioxidant GSH system and its related enzymes and not less important slowing down the progressive neuronal death.

In the present review, we describe the complex network of molecular mechanisms underpinning acute and chronic neurodegeneration, focusing on the disturbance in redox homeostasis, as a common mechanism behind five pivotal risk factors: aging, oxidative stress, inflammation, glycation, and vascular injury. Aging is the primary unchangeable risk factor, and it is characterized by an extensive stress condition that enhances the loss of homeostasis, involving in particular the immune and inflammatory responses, which, in turn, increases oxidative stress generating a vicious circle [58].

Considering the complex multifactorial nature of neurodegenerative diseases, a preventive strategy able to simultaneously target multiple risk factors and disease mechanisms at an early stage is most likely to be effective to slow/halt the progression of neurodegenerative diseases. The holistic approach to neurodegeneration in the present review, taking into account and integrating several common risk factors, will provide critical insights that will most likely contribute to significant advances in the quest for new preventive pharmacological strategies to neurodegenerative disorders.
